# The Role of Individual Disulfide Bonds of μ-Conotoxin GIIIA in the Inhibition of Na_V_1.4

**DOI:** 10.3390/md14110213

**Published:** 2016-11-18

**Authors:** Penggang Han, Kang Wang, Xiandong Dai, Ying Cao, Shangyi Liu, Hui Jiang, Chongxu Fan, Wenjian Wu, Jisheng Chen

**Affiliations:** 1 College of Science, National University of Defense Technology, Changsha 410073, Hunan, China; hanpeng1021@126.com (P.H.); wjwu67@126.com (W.W.); chenjsh@cae.cn (J.C.); 2 Beijing Institute of Pharmaceutical Chemistry, Beijing 102205, China; yiyongjun1949@163.com (K.W.); daixiandong@139.com (X.D.); cao_ying2002@hotmail.com (Y.C.); shangyiliu@sohu.com (S.L.); jiangtide@sina.cn (H.J.)

**Keywords:** μ-conotoxin, GIIIA, disulfide bond, Na_V_1.4, electrophysiology

## Abstract

μ-Conotoxin GIIIA, a peptide toxin isolated from *Conus geographus*, preferentially blocks the skeletal muscle sodium channel Na_V_1.4. GIIIA folds compactly to a pyramidal structure stabilized by three disulfide bonds. To assess the contributions of individual disulfide bonds of GIIIA to the blockade of Na_V_1.4, seven disulfide-deficient analogues were prepared and characterized, each with one, two, or three pairs of disulfide-bonded Cys residues replaced with Ala. The inhibitory potency of the analogues against Na_V_1.4 was assayed by whole cell patch-clamp on rNa_V_1.4, heterologously expressed in HEK293 cells. The corresponding IC_50_ values were 0.069 ± 0.005 μM for GIIIA, 2.1 ± 0.3 μM for GIIIA-1, 3.3 ± 0.2 μM for GIIIA-2, and 15.8 ± 0.8 μM for GIIIA-3 (-1, -2 and -3 represent the removal of disulfide bridges Cys3–Cys15, Cys4–Cys20 and Cys10–Cys21, respectively). Other analogues were not active enough for IC_50_ measurement. Our results indicate that all three disulfide bonds of GIIIA are required to produce effective inhibition of Na_V_1.4, and the removal of any one significantly lowers its sodium channel binding affinity. Cys10–Cys21 is the most important for the Na_V_1.4 potency.

## 1. Introduction

Conotoxins are small, cysteine-rich bioactive peptides derived from the venom of tropical marine cone snails, genus *Conus*. Conotoxins have a wide range of pharmacological targets, including different isoforms of ion channels and membrane receptors [1]. Thus, conotoxins are useful research tools and promising drug leads for diseases related to these targets. Ziconotide, ω-conotoxin MVIIA, has been approved by the US Food and Drug administration as a local analgesic for severe chronic pain treatment [2], and several other pain relief drugs based on conotoxins are under preclinical or clinical testing [3,4].

**Figure 1:**
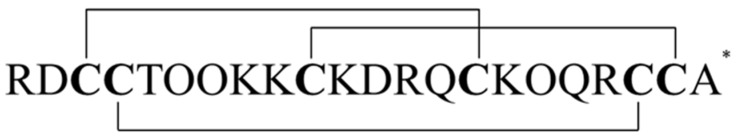
Amino acid sequence and disulfide bonds of μ-conotoxin GIIIA. *: amidated *C*-terminus; O: trans-4-hydroxyproline.

μ-Conotoxins belong to the M superfamily of conopeptides and comprise 16 to 26 residues with six Cys residues arranged in a class III framework (–C1C2–C3–C4–C5C6–) [5]. μ-Conotoxins are the only venom peptides that selectively inhibit voltage-gated sodium channels by sterically and electrostatically blocking the ion flux via binding to the outer vestibule of the channel [6]. The μ-conotoxin GIIIA isolated from *Conus geographus* was among the first to be characterized. GIIIA consists of 22 amino acids (see Figure 1) packed in a pyramidal structure, with three disulfide bonds and three hydroxyproline residues [7]. GIIIA preferentially blocks skeletal muscle sodium channel subtype Na_V_1.4 by a rather complex toxin–pore interaction, with several residues in the toxin contributing to the high-affinity binding [8].

The most challenging step in the chemical synthesis of disulfide-rich peptides, such as μ-conotoxin, is oxidative folding, which is often time-consuming, low in efficiency and poor in yield [9]. The disulfide bonds in the peptide sequences give rise to a well-defined and constrained framework and generally play an important role in their biological action [10]. However, growing evidence suggests that many disulfide-rich peptides do not need all their native disulfide bonds to retain biological activity [11,12,13]. For example, the removal of the Cys1–Cys16 bridge in ω-conotoxin MVIIA did not abolish its calcium channel binding affinity [14]. The deletion of a disulfide bond can no doubt accelerate the chemical synthesis of the small neuropeptide and benefit the structure-function studies.

As a valuable probe for sodium channel research, GIIIA has attracted considerable attention. Previous studies have mainly focused on the key residues in the toxin-channel interaction, but the role of individual disulfide bonds in the inhibition of Na_V_1.4 remains ambiguous. In this study, we assessed the contributions of the three individual disulfide bonds to the inhibitory activity against Na_V_1.4. GIIIA and seven analogues, each with one, two, or three pairs of disulfide-bonded Cys residues replaced with Ala, were prepared, and their blocking potency was assayed using the whole cell patch-clamp technique on HEK293 cells transiently expressing sodium channel subtype rNa_V_1.4.

## 2. Materials and Methods

### 2.1. Materials and Apparatus

Rink amide resins, Fmoc-protected amino acids, and other reagents for peptide synthesis were purchased from GL Biochem, Shanghai, China. HPLC-grade ACN was obtained from Sigma-Aldrich, St. Louis, MO, USA. Lipofectamine 2000 was purchased from Life Technologies, Chagrin Falls, OH, USA. All reagents used were analytical grade. The HEK293 cell strain was offered by the Cell Center of Chinese Academy of Sciences, Shanghai, China. Standard μ-conotoxin GIIIA was purchased from Bachem, Bubendorf, Switzerland.

Solid-phase peptide synthesis was performed on a CEM Liberty peptide synthesizer (CEM, Matthews, NC, USA). MALDI-TOF-MS was measured on a Bruker ultraflex TOF/TOF mass spectrometer (Bruker Daltonics, Billerica, MA, USA) with α-cyano-4-hydroxycinnamic acid as the matrix. Reversed-phase HPLC was performed on an Agilent 1100 system with a dual wavelength UV detector (Agilent, Santa Clara, CA, USA). C18 Vydac columns were purchased from Grace, Deerfield, IL, USA. Whole cell patch-clamp recordings were conducted on an Axon700B amplifier (Molecular Devices, Sunnyvale, CA, USA). The MF-900 Microforge and Shutter P-97 Micropipette puller were the products of Narishige Group, Tokyo, Japan and Sutter Instrument, Novato, CA, USA, respectively.

### 2.2. Chemical Synthesis of Peptides

Peptides were synthesized on Rink amide resin using Fmoc chemistry and standard side chain protection except on the cysteine residues. To prepare GIIIA analogues containing two disulfide bonds, the peptides were synthesized using a selective Cys protection scheme. One pair of connected Cys residues was protected by trityl groups, and the other pair was protected by Acm groups. For GIIIA and other analogues, all Cys residues were trityl-protected.

Following chain assembly, the peptides were partially deprotected and cleaved from the resin with a mixture of TFA/TIS/H_2_O (95:2.5:2.5, by volume) for 2 h at room temperature. The cleaved peptides were filtered, precipitated with cold ethyl ether, and washed several times with cold ethyl ether. The released peptides were purified by reversed-phase HPLC on a semi-preparative C18 Vydac column (218TP510, 10 mm × 250 mm) eluted with a gradient of 0%–50% ACN in 0.1% TFA over 50 min. The flow rate was 3 mL/min, and absorbance was monitored at 214 nm. The masses of the linear peptides were validated by MALDI-TOF MS.

### 2.3. Oxidative Folding of Analogues Containing Two Disulfide Bonds

The purified linear peptides contain two Acm-protected Cys and two free thiols. A two-step oxidation protocol was used to selectively fold the peptides. To promote disulfide bond formation between the free thiols, the peptides were diluted to a final concentration of 0.2 mg/mL with 0.2 M aqueous ammonium bicarbonate (pH 7.8) and stirred moderately at room temperature. Oxidation reactions were analyzed by HPLC and judged to be complete when the HPLC profiles reached a steady equilibrium state with one main peak. After the first oxidation step, the peptides were concentrated and purified by semi-preparative HPLC as described above.

To remove the Acm groups from the other pair of cysteines and close the remaining disulfide bridge, the monocyclic peptides were treated with 2 mM iodine dissolved in 50% aqueous ACN containing 0.1% TFA. The progress of Acm-deprotection and subsequent disulfide bond formation was monitored by analytical HPLC at regular intervals until completion (approximately 30 min). The reaction was then quenched by addition of a 5 mg/mL ascorbic acid solution, resulting in decolorization of the solution. The fully oxidized peptides were then subjected to semi-preparative HPLC.

The purities of the folded peptides were assessed by analytical HPLC using a C18 Vydac column (218TP54, 4.6 mm × 250 mm) with a gradient of 0%–30% ACN in 0.1% TFA in 30 min at 1 mL/min. GIIIA and other analogues were oxidized by one-step air oxidation and purified by HPLC as described above. The identities of all the final products were confirmed by MALDI-TOF MS.

### 2.4. Plasmid Constructions

SCN4A, encoding the α subunit of rNa_V_1.4, was cloned from rat skeletal muscle cDNA and inserted into the multiple cloning sites of the pcDNA3.1^+^ vector using a PCR-based strategy. The pcDNA3.1^+^-rNa_V_1.4 plasmid was purified and confirmed by DNA sequencing analysis. The pcDNA3.1^+^-EGFP plasmid was constructed similarly.

### 2.5. Cell Culture and Transient Expression in HEK293 Cells

HEK293 cells were cultured in Dulbecco’s Modified Eagle’s Medium supplemented with 10% fetal calf serum and 1% penicillin-streptomycin. Cells were maintained at 37 °C in a humidified incubator with 95% air and 5% CO_2_. HEK293 cells were trypsinized, diluted with culture medium, and grown in 35-mm culture dishes. When grown to >60% confluence, cells were transfected with a 5:1 ratio of pcDNA3.1^+^-rNa_V_1.4 and pcDNA3.1^+^-EGFP using the Lipofectamine 2000 transfection kit. Approximately 24 h after transfection, cells displaying green fluorescence were selected for electrophysiological recordings.

### 2.6. Electrophysiology

Whole cell patch-clamp recordings from HEK293 cells were performed using an Axon700B amplifier controlled by a pClamp10.2 data acquisition system [15,16]. For the recordings, the cells were perfused with extracellular solution containing 140 mM NaCl, 4 mM KCl, 1 mM MgCl_2_, 2 mM CaCl_2_, 5 mM d-glucose, and 10 mM HEPES. The pH of the solution was adjusted to 7.4 using NaOH. Patch pipettes had resistances of 2–4 MΩ, and the series resistance was compensated for by approximately 60% to minimize voltage errors. The pipettes were filled with intracellular solution containing 50 mM CsCl, 10 mM NaCl, 60 mM CsF, 2 mM MgCl_2_, 20 mM EGTA, and 10 mM HEPES with the pH adjusted to 7.2 by the addition of CsOH. Stock solutions of GIIIA and the analogues were diluted to the final concentrations in extracellular solution and applied through the perfusion system. Sodium currents were evoked by a depolarization pulse from a holding potential (−80 mV) to a test voltage (−10 mV). The pulse duration was 50 ms with an interval of 5 s. Output signals were low-pass filtered at 5 kHz and sampled at 10 kHz. All experiments were performed at constant temperature, 21–24 °C.

Individual IC_50_ values were calculated by fitting dose–response data to the following Hillslope equation:

I/I_max_ = 1/(1 + ([toxin]/IC_50_) ^ Hillslope)

where Imax and I are the peak sodium currents in the absence and presence of toxin, [toxin] is the drug concentration, and Hillslope is the fitting coefficients.

Electrophysiological data were analyzed using the Clampfit10.2 (Molecular Devices, Sunnyvale, CA, USA) and Sigmaplot10.0 (Systat Software, Inc., San Jose, CA, USA) software. Data were presented as the mean ± SEM with *n* = number of independent measurements. Datasets were tested for statistical significance using the Student’s *t* test, with *p* < 0.05 representing significance.

## 3. Results

### 3.1. Chemical Synthesis and Oxidative Folding

To assess the contributions of individual disulfide bonds of GIIIA to the inhibition of Na_V_1.4, seven analogues, each with one, two, or three pairs of disulfide-bonded Cys residues replaced with Ala, were synthesized, as shown in Table 1. The three disulfide bonds (Cys3–Cys15, Cys4–Cys20, Cys10–Cys21) of GIIIA [7] were numbered 1, 2 and 3. The analogues are named GIIIA-number(s), in which each number represents that the corresponding disulfide bond was removed. For example, GIIIA-3 represents the analogue missing the third disulfide bond (Cys10–Cys21).

**Figure 2:**
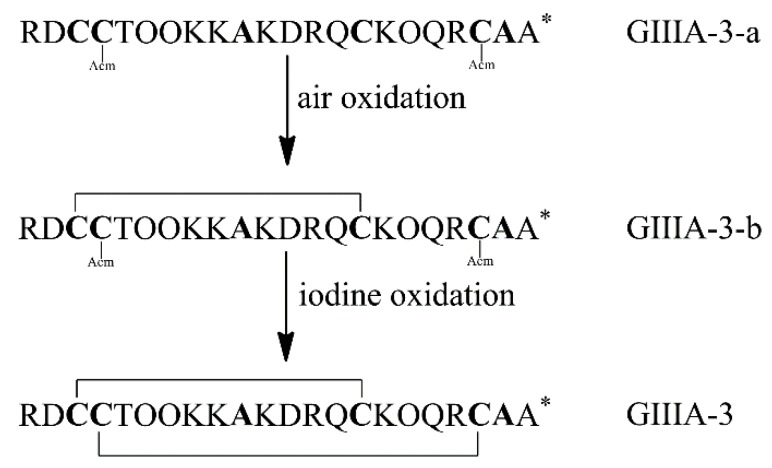
Synthesis and oxidation strategy for GIIIA-3. GIIIA-3 was chemically synthesized using two sets of Cys thiol-protecting groups. Cys3 and Cys15 were protected with trityl groups, while Cys4 and Cys20 were protected with Acm groups. A two-step oxidation protocol was used to selectively fold the linear peptide. Air oxidation was applied to form the disulfide bond Cys3–Cys15, and iodine oxidation was used to remove Acm protection and close the second disulfide bond Cys4–Cys20. * denotes *C*-terminal amide.

**Figure 3:**
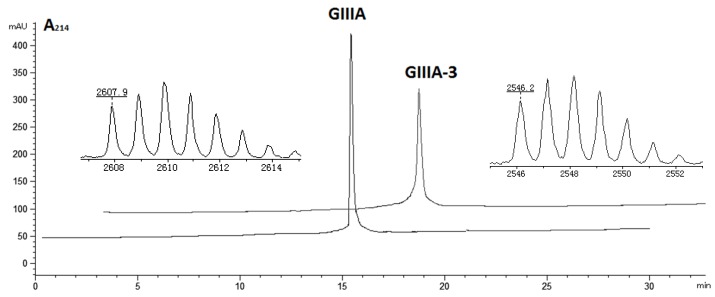
HPLC elution profiles and MALDI-TOF mass spectra of GIIIA and GIIIA-3. Final folded peptides were dissolved in 0.1% TFA, then applied to a C18 analytical column and eluted with a linear gradient of 0%–30% ACN in 0.1% TFA at 1 mL/min over 30 min. Peaks were collected for MALDI-TOF-MS analysis. The MS data (MH^+^, 2607.9 for GIIIA, 2546.2 for GIIIA-3) were consistent with the calculated mass, indicating that the peptides were correctly synthesized.

The linear forms of the analogues were chemically synthesized with an automated peptide synthesizer using Fmoc-based protocols, as described in Materials and Methods. The Rink amide resin was used as a solid support, on which the first amino acid was loaded. The linear peptides prepared by reversed-phase HPLC were characterized by mass spectrometry to determine their identity. For analogues containing two disulfide bonds, a strategy combining orthogonal cysteine protection with two-step oxidation was adopted to ensure the correct formation of the native disulfide bridges [17], as illustrated for GIIIA-3 in Figure 2. Formation of the first disulfide bridge was accomplished by ammonium bicarbonate-mediated air oxidation, whereas the second bridge was closed using iodine. GIIIA and remaining analogues were folded directly by air oxidation. The final products were purified by HPLC and confirmed by MALDI-TOF-MS with GIIIA: *m*/*z* 2606.9 (calculated = 2607.2), GIIIA-1/GIIIA-2: *m*/*z* 2545.1 (calculated = 2545.2), GIIIA-3: *m*/*z* 2545.2 (calculated = 2545.2), GIIIA-12: *m*/*z* 2483.1 (calculated = 2483.3), GIIIA-13: *m*/*z* 2483.3 (calculated = 2483.3), GIIIA-23: *m*/*z* 2483.2 (calculated = 2483.3), GIIIA-123: *m*/*z* 2422.3 (calculated = 2422.3). GIIIA was identical to the standard GIIIA according to HPLC analysis. Figure 3 shows the HPLC elution profiles and corresponding mass spectra of GIIIA and GIIIA-3.

### 3.2. Effect of the Analogues on rNa_V_1.4 Expressed in HEK293 Cells

It has been demonstrated that GIIIA is a selective blocker of the skeletal muscle subtype Na_V_1.4 [7]. Therefore, we assessed the biological activity of seven disulfide-deficient analogues by measuring their potency for blocking rNa_V_1.4, expressed in HEK293 cells by whole cell patch-clamp protocols. Control experiments were performed on more than 100 cells not transfected with rNa_V_1.4 expressing plasmids, and no Na^+^ currents were detected.

**Figure 4:**
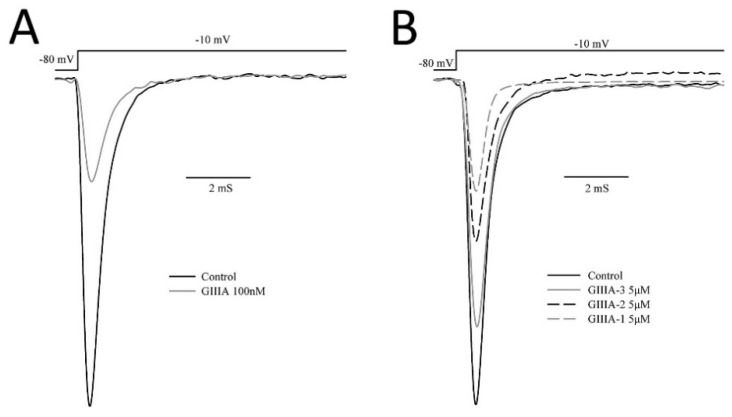
Effect of GIIIA and its analogues on rNa_V_1.4 expressed in HEK293 cells. Sodium currents were elicited by a depolarizing step from a holding potential of −80 mV to a test voltage of −10 mV from HEK293 cells expressing rNa_V_1.4. Representative current traces are shown in the absence (Control) and presence of (**A**) 100 nM GIIIA; (**B**) 5 μM GIIIA-1, GIIIA-2, GIIIA-3.

Representative current traces in the absence and presence of 100 nM GIIIA, 5 μM GIIIA-1, GIIIA-2, GIIIA-3 are shown in Figure 4. The three analogues inhibit the Na^+^ currents in a similar way to GIIIA. The remaining currents do not show any obvious alterations in kinetics.

**Figure 5:**
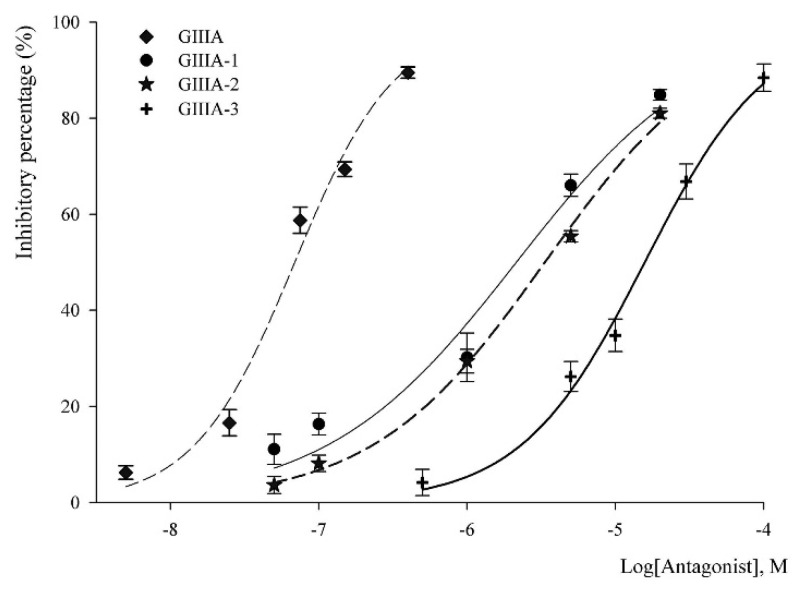
Concentration–response curves obtained for the inhibition of rNa_V_1.4-mediated Na^+^ currents by GIIIA (◆), GIIIA-1(●), GIIIA-2 (★) and GIIIA-3 (╋). Corresponding IC_50_ values were 0.069 ± 0.005 μM for GIIIA, 2.1 ± 0.3 μM for GIIIA-1, 3.3 ± 0.2 μM for GIIIA-2, and 15.8 ± 0.8 μM for GIIIA-3. Values are mean ± SEM from three to five separate cells.

The inhibitory potency of the analogues was measured at various concentrations, and the electrophysiological data were fitted to the Hillslope equation, yielding corresponding IC_50_ values, as listed in Table 2. GIIIA inhibits the depolarization-evoked Na_V_1.4 currents in a concentration-dependent manner (Figure 5) with an IC_50_ of 0.069 ± 0.005 μM, similar to the values reported previously [18,19]. GIIIA-12, GIIIA-13, and GIIIA-23, which lost two disulfide bonds, and the linear peptide GIIIA-123, did not exhibit any significant blockade at 100 μM, suggesting that they cannot adopt an appropriate conformation to inhibit the sodium channel. The potency of the analogues containing two of three disulfide bonds against Na_V_1.4 decreased in the following order: GIIIA-1 > GIIIA-2 > GIIIA-3. The corresponding IC_50_ values were 2.1 ± 0.3 μM for GIIIA-1, 3.3 ± 0.2 μM for GIIIA-2, and 15.8 ± 0.8 μM for GIIIA-3, indicating that among the three individual disulfide bridges, Cys10–Cys21 is the most important for the blockade of Na_V_1.4, while Cys3–Cys15 is the least important. However, the most potent analogue, GIIIA-1, missing the Cys3–Cys15 disulfide bridge, still reduced the inhibition of Na_V_1.4 by more than 30-fold, and the removal of Cys10–Cys21 reduced the potency approximately 200-fold. This result suggests that all three disulfide bonds of GIII A are needed to produce effective inhibition of Na_V_1.4, and the specific conformation stabilized by the three disulfide bonds is essential for the blockade.

## 4. Discussion

This research investigated the role of individual disulfide bonds of GIIIA in the inhibition of Na_V_1.4. Our data demonstrate that all three disulfide bonds are important in forming the specific conformation of the molecule for exerting the biological activity, and depleting any one would significantly reduce its Na_V_1.4 potency. In particular, the removal of the Cys10–Cys21 bridge produced an approximately 200-fold loss in binding affinity for Na_V_1.4.

Mutational studies on GIIIA have proven that Arg13 is a key residue for the blockade of channel function [20,21,22]. Although Arg13 plays a prominent role, it has been verified that other residues including Arg1, Lys11, Gln14, Lys16, Hyp17 and Arg19 also affect the bioactivity [23,24,25]. Recently, homology models of mammalian Na_V_1.4 based on the X-ray crystal structures of several bacteria voltage-gated sodium channels were constructed to study GIIIA binding to Na_V_1.4 [26,27]. The binding mode of the GIIIA-Na_V_1.4 complex indicates that the toxin blocks the pore through multiple interactions of Arg13, Lys16, and Arg19 with the acidic residues in the channel vestibule [26,27,28]. We deduce that the rigidity of GIIIA maintained by three disulfide bonds are important for Na_V_1.4 inhibition. The removal of any one disulfide bridge would cause a loss of rigidity, reducing the affinity of the peptide to the pore.

Na_V_1.4 blockade by different disulfide isomers of μ-conotoxin PIIIA was investigated [29]. Tietze and coworkers [8] found that three disulfide-bridged peptide isomers, termed PIIIA-1, -2 and -3, were able to block Na_V_1.4 with considerable, yet distinguishable potency. PIIIA-1, with 1–5/2–4/3–6 connectivity, was twice as potent as PIIIA-2, which adopted the same 1–4/2–5/3–6 pattern as PIIIA [30]. PIIIA-3, with 1–2/3–4/5–6 connectivity, retained about 50% activity of PIIIA. NMR analysis and molecular dynamic simulations revealed that the key residue R14 of PIIIA-1 binds much deeper in the channel pore than PIIIA and PIIIA-3 [29]. This result offered straightforward proof that disulfide bridges affect the docking of amino acid residues to the pore. Moreover, consistently with our results, the reduced PIIIA was not active [29]. This finding also suggested that the rigid structure maintained by three disulfide bridges is necessary for the sodium channel block.

In another study, the contributions of individual disulfide bridges in μ-conotoxin BuIIIB to Na_V_1.3 inhibition were assessed [31]. BuIIIB shares the 1–4/2–5/3–6 disulfide pattern with the following disulfide bridges: Cys5–Cys17 (1–4), Cys6–Cys23 (2–5), and Cys13–Cys24 (3–6) [31,32]. In contrast to GIIIA, the study suggested that the removal of the Cys5–Cys17 bridge reduced the blockade potency by 80-fold, whereas removal of either the Cys6–Cys23 or Cys13–Cys24 bridges caused only a slight impact on affinity. This result indicated that the Cys5–Cys17 bridge in BuIIIB is the most important disulfide bridge for Na_V_1.3 inhibition [31]. The systematic study of the binding modes of GIIIA and BuIIIB to the Na_V_1 channels reveals a common binding motif for the blockade [28]. The binding motif consists of three corresponding basic residues that form contacts with the channel pore [28]. Intriguingly, despite sharing a common motif and same disulfide pattern, the roles of the individual disulfides bridges of GIIIA and BuIIIB in the Na_V_1 inhibition are different. This finding may be attributed to the differences in their tertiary structure.

In conclusion, our study clearly shows that all three disulfide bonds in GIIIA are needed to produce effective inhibition of Na_V_1.4 and validates the important contribution of individual disulfide bonds to toxin-Na_V_1.4 interactions. These results will provide valuable information, making it possible to elucidate the mechanisms of GIIIA binding to the sodium channel Na_V_1.4 and design analogues with better affinity and selectivity for therapeutic use.

## Figures and Tables

**Table 1:** Sequence and cysteine frame of GIIIA and seven analogues.

Peptide ^#^	Sequence	Cysteine Frame
GIIIA	RDCCTOOKKCKDRQCKOQRCCA *	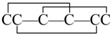
GIIIA-1	RDACTOOKKCKDRQAKOQRCCA *	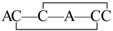
GIIIA-2	RDCATOOKKCKDRQCKOQRACA *	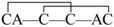
GIIIA-3	RDCCTOOKKAKDRQCKOQRCAA *	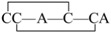
GIIIA-12	RDAATOOKKCKDRQAKOQRACA *	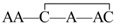
GIIIA-13	RDACTOOKKAKDRQAKOQRCAA *	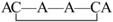
GIIIA-23	RDCATOOKKAKDRQCKOQRAAA *	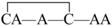
GIIIA-123	RDAATOOKKAKDRQAKOQRAAA *	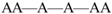

^#^: The three disulfide bonds (Cys3–Cys15, Cys4–Cys20, Cys10–Cys21) of GIIIA are numbered 1, 2 and 3. The analogues are named GIIIA-number(s), in which each number represents that the corresponding disulfide bond was removed. * represents an amidated *C*-terminus.

**Table 2:** The inhibitory potency of GIIIA and its analogues against rNa_V_1.4.

Peptides	IC_50_ (μM) *	Inhibitory Potency
GIIIA	0.069 ± 0.005	1
GIIIA-1	2.1 ± 0.3	0.033
GIIIA-2	3.3 ± 0.2	0.021
GIIIA-3	15.8 ± 0.8	0.005
GIIIA-12	>100	<0.0007
GIIIA-13	>100	<0.0007
GIIIA-23	>100	<0.0007
GIIIA-123	>100	<0.0007

* Data are mean ± SEM from at least three cells.
